# Design of a Tunable Absorber Based on Active Frequency-Selective Surface for UHF Applications

**DOI:** 10.3390/ma12233989

**Published:** 2019-12-02

**Authors:** Kainan Qi, Liangsheng Li, Jianxun Su, Yongqiang Liu, Junwen Chen

**Affiliations:** 1 College of Information Engineering, Communication University of China, Beijing 100854, China; sujianxun_jlgx@163.com (J.S.); lixiaoshen@chinasarft.gov.cn (J.C.); 2 Science and Technology on Electromagnetic Scattering Laboratory, Beijing 100854, China; liliangshengbititp@163.com (L.L.); liuyq1990@126.com (Y.L.)

**Keywords:** tunable absorber, active frequency-selective surface, ultrahigh frequency, varactors

## Abstract

An ultrathin tunable absorber for the ultrahigh frequency (UHF) band is presented in this paper. The absorber is a single-layer structure based on the topology of a Salisbury screen, in which the conventional resistive layer is replaced by an active frequency-selective surface (AFSS) loaded with resistors and varactors. The reflectivity response of the absorber can be controlled by adjusting the reverse bias voltage for the varactors, which is verified by both simulated and measured results. The experimental results show that the reflectivity response of the absorber can be modulated below −10 dB over a frequency band ranging from 415 to 822 MHz. The total thickness of the absorber, 10 mm, is equivalent to only *λ*/72 of the lower limit frequency. The absorbing mechanism for the designed absorber is illustrated by simulating the volume loss density distributions. A detailed analysis is also carried out on the basis of these parameters, such as the AFSS shape, resistor, thickness of the foam, thickness and permittivity of the dielectric substrate, and incident angles, which contribute to the reflectivity of the AFSS absorber.

## 1. Introduction

Stealth technology is one of the most important military technologies and is of concern to all nations. Radar-absorbing materials (RAM) can effectively reduce the radar cross section (RCS) [1] of aircrafts and are commonly used in stealth missions. Traditional absorbers [2,3,4] are well used at high frequencies above 2 GHz. However, low-frequency absorbers are also in great demand.

As radar detection equipment extends to the near-meter wavelength regime, high-performance absorbers are required at lower frequencies, especially in the ultrahigh frequency (UHF) band. In addition, low-frequency absorbing materials can also be used for electromagnetic compatibility (EMC) [5], radio frequency identification (RFID) [6], and sub-GHz wireless systems [7].

Metamaterial absorbers (MMA) have attracted much attention in recent years [8,9,10,11,12,13,14], after a perfect MMA with near unity absorption in microwave regime was first reported by Landy et al. [15]. MMA are also used for low frequency applications [16,17,18,19]; for example, Khuyen et al. [20] proposed an ultrathin polarization-insensitive metamaterial absorber, which exhibits a peak absorption of 97% at 250 MHz. Zuo et al. [21] presented a wideband metamaterial absorber using a metallic incurved structure, which has an absorptivity of more than 90% at 0.8–2.7 GHz.

Rozanov [22] discussed the problems of the ultimate thickness-to-bandwidth ratio of a radar absorber, observing that passive absorbers are usually very thick or have a narrow absorption bandwidth below 2 GHz. This problem could be solved by using a tunable absorber, the electromagnetic characteristics of which can be dynamically modulated. There are several methods to make a tunable absorber, including those based on the use of graphene [23], liquid crystals [24], superconductors [25], conducting polymers [26], an active frequency-selective surface (AFSS) [27], and so on. In terms of cost and response time, AFSS was adopted in this study to design the tunable absorber. The tunable absorber based on AFSS [28,29,30,31,32] has an ultrathin layered structure, which can be used to simultaneously control its reflection response to obtain a wide absorbing bandwidth. For instance, Mias et al. [33] designed a tunable microwave absorber based on a high-impedance surface and presented data showing that the reflectivity response of the absorber can be controlled over the frequency band from 1.72 to 1.93 GHz. Zhao et al. [34] designed a tunable metamaterial absorber using varactor diodes, which had a tunable bandwidth of 1.5 GHz and an absorption rate of more than 90% when the bias voltage changed from 0 to −19 V. However, these works cannot simultaneously guarantee both tunable absorption bandwidth and material thickness.

In this study, we designed a thin absorber with a sufficiently large tunable bandwidth. In this design, the resistors and varactors are embedded between adjacent resonant units, and the absorbed frequency can be tuned continuously by adjusting the bias voltage of varactors. The experimental results validate the tunability of the designed absorber. The tunability ranges from 415 to 822 MHz, with a reflectivity below −10 dB and a thickness of 10 mm that corresponds to only *λ*/72 of the resonance frequency. This work is important for stealth and other microwave applications in the future.

## 2. Design, Simulations, and Experiments

### 2.1. Structure of Proposed Absorber

**Figure 1:**
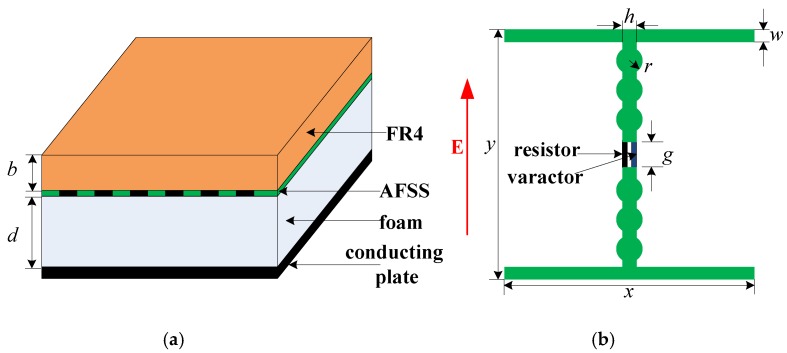

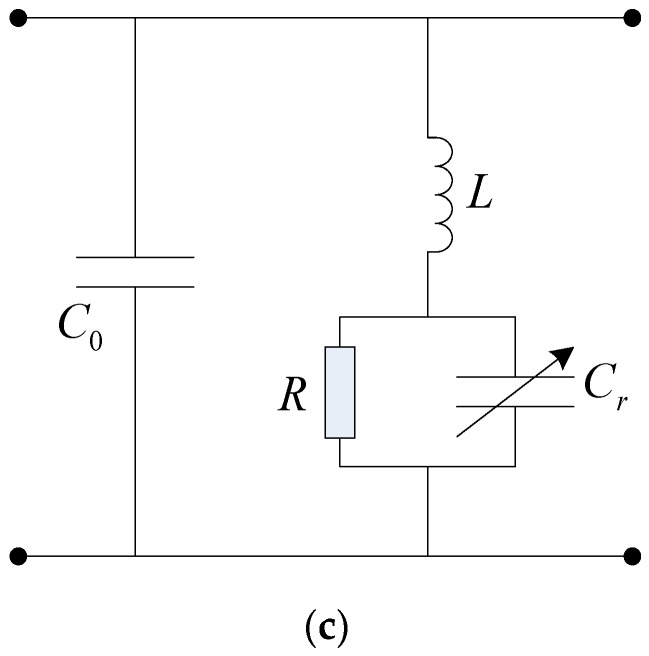
The designed absorber: (**a**) three-dimensional (3D) structure, (**b**) an active frequency-selective surface (AFSS) unit cell, and (**c**) the equivalent circuit model of the AFSS.

Figure 1 shows the structure of the proposed AFSS absorber. The top layer is a FR4 dielectric substrate, which has a relative permittivity of 4.4(1-j0.02) and a thickness of 1 mm. The next layer is the AFSS, which is loaded with resistors and varactors and printed onto the FR4 substrate; the thickness of the copper is 0.018 mm. The third layer, used as an independent layer, is a 9 mm-thick foam with very low dielectric loss ($\varepsilon_{r}$ = 1.05, tanδ = 0.002). The bottom layer is a metal ground. Figure 1b shows the geometric structure of the unit cell, which is based on a dipole. It is composed of two bias lines along the x-axis and two strips with six circles along the y-axis. The final geometric dimensions are given as: x = y = 50 mm, h = 3 mm, r = 3 mm, g = 2 mm, w = 1 mm, b = 1 mm, and d = 9 mm. A varactor and a resistor are loaded onto the gap between the two strips.

### 2.2. Equivalent Circuit Model of the Proposed Absorber

The equivalent circuit model for the AFSS layer is shown in Figure 1c. A varactor with a variable capacitance C_r_ is connected in parallel to a resistor R. L and C_0_ are the distributed parameters generated by the topological structure of the unit cell pattern. C_0_ is relative to the gap between the two bias lines and can be ignored. The whole impedance of the AFSS is given by

(1) $$Z_{AFSS}=\frac{1}{j\omega L+\frac{1}{j\omega L+\frac{1}{j\omega C_{r}+\frac{1}{R}}}}\approx j\omega L+\frac{1}{j\omega C_{r}+\frac{1}{R}}=j\omega L+\frac{R}{1+j\omega RC_{r}}.$$

The resonant frequency of the AFSS is determined by

(2) fr=1(2πLCr).

**Figure 2:**
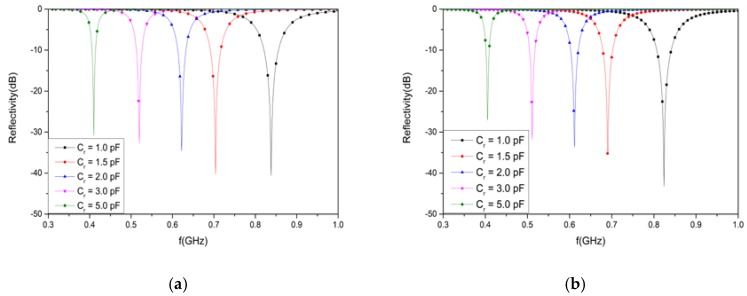
The simulated results for varying *C_r_*: (**a**) by the equivalent circuit method (ECM) or (**b**) by a high frequency structure simulator (HFSS).

The AFSS absorber was simulated by MATLAB based on an equivalent circuit method (ECM). A plane wave normally occurred on the absorber, with the electric field polarized along the y-axis, as shown in Figure 1b. R is the resistance of the lumped resistor. *C_r_* is the capacitance of the varactor, which increases as the reverse bias voltage decreases. Figure 2a shows the simulated reflectivity with a varying *C_r_* and fixed R = 2400 Ohms. In this figure, the resonance frequency decreases as C_r_ increases, and the reflectivity nadir is controlled over a wide frequency band from 410 to 835 MHz when C_r_ is varied from 1 to 5 pF.

The AFSS absorber was numerically simulated by a high-frequency structure simulator (HFSS) in order to verify the equivalent circuit model. In Figure 2b, the minimum value of the reflectivity is modulated over a wide frequency band from 406 to 824 MHz when C_r_ varies from 1 to 5 pF. The numerical simulation suggests that the numerical solution approaches the theoretical solution very well.

### 2.3. Experiment and Results

**Figure 3:**
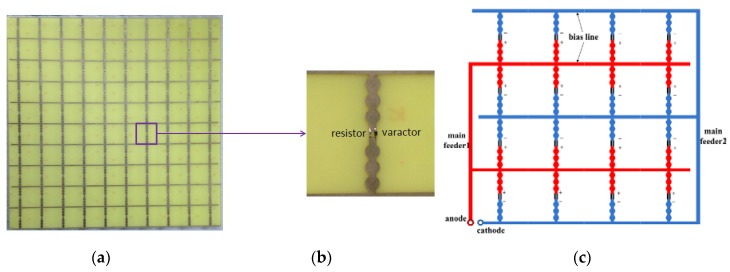
Photograph of the fabricated AFSS prototype: (**a**) the whole AFSS array, (**b**) the unit cell, and (**c**) a schematic diagram of the bias circuit.

A sample of the proposed absorber was fabricated based on the design shown in Figure 1. The AFSS pattern was constructed on a FR4-printed circuit board by standard photoetching techniques and was loaded with resistors and varactors (NXP, BB131) by manual welding techniques. A photograph of the AFSS prototype is shown in Figure 3a, and Figure 3b shows the topological structure of the unit cell. The AFSS board measures 500 by 500 mm and contains 100 (10 × 10) dipole elements. The FR4 substrate has a relative permittivity of 4.4 (1-j0.02) and a thickness of 1 mm. The AFSS prototype was pasted with a foam board with a thickness of 9 mm, so the total thickness of the absorber sample was 10 mm. Figure 3c shows a schematic diagram of the circuit connection in the AFSS array. An element was divided into two parts by devices. The adjacent two subunits were connected back to back, together vertically, and then, a row of units was connected together by a bias line. Finally, the red array was connected to the anode by the main feeder 1, and the blue array was connected to the cathode through the main feeder 2. The adjacent two rows of varactors were welded with the opposite polarity to ensure that the same polarity was connected together. As a result, all the devices were in parallel and biased at the same voltage.

**Figure 4:**
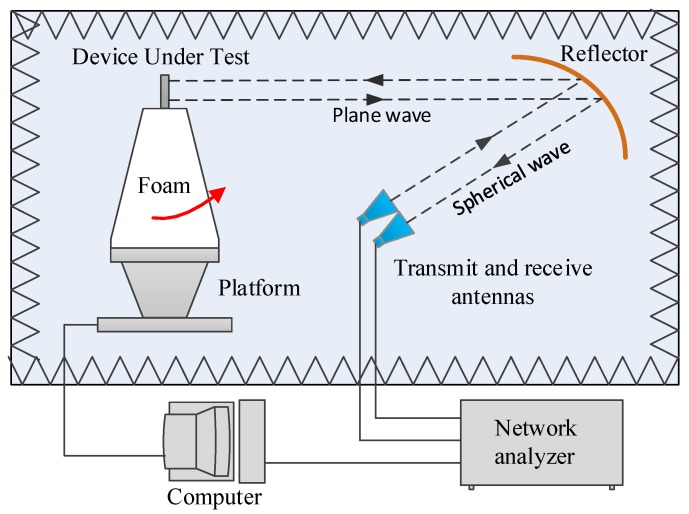
Schematic view of the compact range system for the reflectivity measurement.

**Figure 5:**
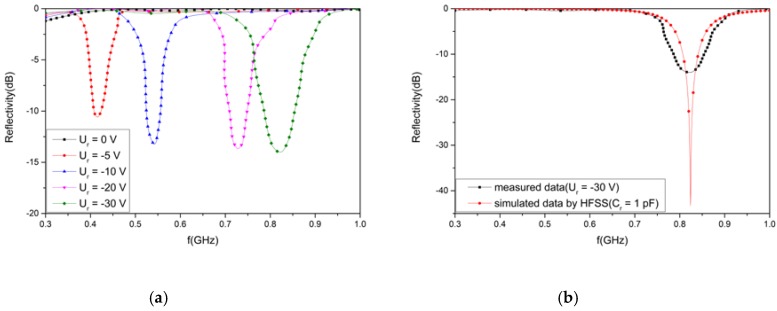
(**a**) Measured results for the absorber with various voltages; (**b**) the simulated and measured absorption performance.

The reflectivity of the proposed absorber over the frequency range of 300–1000 MHz for various bias voltages was measured in the anechoic chamber of the Science and Technology on Electromagnetic Scattering Laboratory. The measurement setup for the compact range system is shown in Figure 4. Two identical horn antennas were utilized as transmitting and receiving devices, respectively. The spherical waves emitted by the horn antenna were reflected by a parabolic metal reflector before becoming a plane wave. A short test distance between the sample and reflector was easily realized to meet the far-field conditions. According to the datasheet of BB131, the junction capacitance C_r_ changed from 1 to 5 pF when the reverse bias voltage (*U_r_*) varied from −5 to −30 V. The measured results are presented in Figure 5a. When the bias voltage was 0 V, the AFSS absorber had no absorption. When the inverse bias voltage varied from −5 to −30 V, the resonant frequency moved to a higher frequency and covered a frequency band ranging from 415 to 822 MHz below −10 dB. The total thickness was equivalent to only *λ*/72 of the lower limit frequency and *λ*/36 of the higher limit frequency. The measured results for various reverse voltages (*U_r_*) ranging from −5 to −30 V agree roughly with the simulated results for a varying capacitance (*C_r_*) from 1 to 5 pF. Both the measured and simulated results validate the tunability of the proposed absorber, providing a feasible method for absorption in UHF applications.

Figure 5b shows the simulated and measured absorption performance of the proposed absorber. According to the datasheet of BB131, the capacitance of the varactor is approximately 1 pF when it is biased at −30 V. In Figure 5b, the measured reflectivity is −14.1 dB at 822 MHz, and the reflectivity simulated by the HFSS is −43.2 dB at 824 MHz. It is observed that the resonant frequencies of these two curves agree with each other, but the peak amplitude and bandwidth of the reflectivity are different. This difference occurs, because the size of the experimental sample is limited while being set as an infinite periodic array in simulation. The measurement tolerance exists because of the edge diffraction of the experimental sample and the fabrication tolerance. Moreover, the calculation tolerance exists because of the model complexity of the varactors when simulated by the HFSS.

## 3. Discussion and Analysis

**Figure 6:**
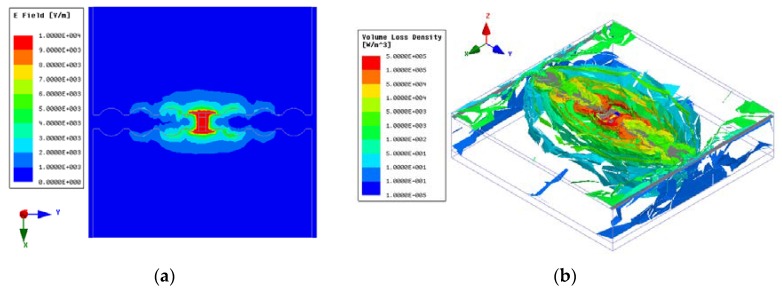
The simulated results obtained by the HFSS: (**a**) the electric field distribution and (**b**) the volume loss density distribution.

The electric field and volume loss density distributions were simulated by using the HFSS at a resonant frequency of 824 MHz with *C_r_* =1 pF to illustrate the absorbing mechanism of the proposed absorber. The field distributions are shown in Figure 6. It is observed that there is a high concentration of electric field around the gap where the resistor and varactor are loaded. According to the volume loss density distribution, the absorber experiences both dielectric and resistive loss, which both contribute to the total energy loss. The loss on the AFSS is mainly caused by absorption from the resistive elements. Resonance occurs at 824 MHz, where the equivalent circuit yields a strong electric field. When R = 2400 Ohms, the input impedance of the absorber matches perfectly with free space, and the incident wave enters inside of the designed structure, generating multiple reflections. Therefore, most of the energy losses arise from absorption of the dielectric substrate, while the paralleled capacitance C_r_ alters the AFSS layer’s impedance and, as a result, the resonant frequency. Hence, the resonance frequency of the absorber can be adjusted.

The reflectivity is a function of certain parameters such as the AFSS shape, resistor, thickness of the foam, thickness and permittivity of the dielectric substrate, and the incident angles. To study the influence of these parameters on the reflectivity, the HFSS was used to carry out simulation analysis for each parameter.

### 3.1. Analysis of the Designed Structure with Different AFSS Shapes

**Figure 7:**
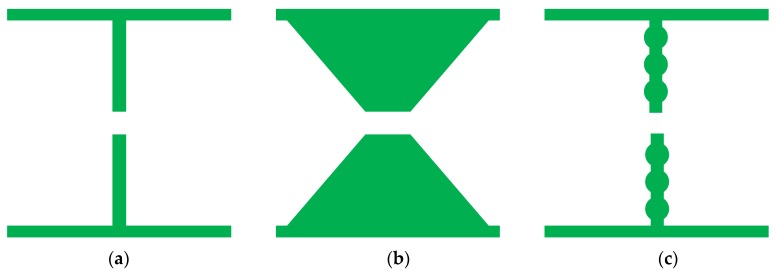
Different shapes for the AFSS unit cell: (**a**) shape 1, (**b**) shape 2, and (**c**) shape 3.

**Figure 8:**
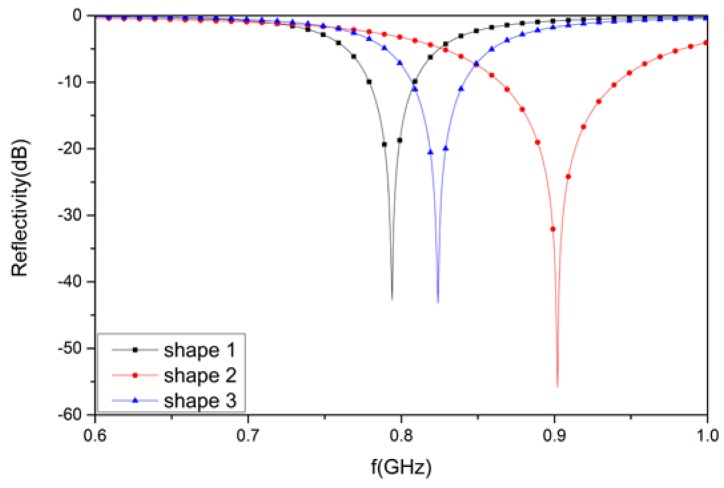
The simulated results for the designed structure with different AFSS shapes.

According to the waveguide handbook [35], L in Equation (2) is mainly determined by the shape of the AFSS element. This can be equivalent to the reactance component and eventually affects the resonance frequency of the designed structure. Figure 7 shows three AFSS cells with different shapes, whose parameters are consistent with Figure 1a. According to the equivalent circuit formula, the inductance L of shape 1 is largest, the L for shape 2 is smallest, and the L for shape 3 lies in the middle. Figure 8 shows the simulated reflectivity for these three AFSS absorbers with R = 2400 Ohms and *C_r_* = 1 pF. The reflectivity nadir of the designed structure is −42.7 dB at 794 MHz for shape 1, −55.9 dB at 902 MHz for shape 2, and −43.2 dB at 824 MHz for shape 3. The relationship between the resonant frequencies for the three shapes is *f_r_*_1_ *<* *f_r_*_3_ *<* *f_r_*_2_.

It is easy to observe that the resonant frequency of the absorber decreases when the equivalent parameter L increases, which is consistent with Equation (2). On the other hand, the value of L affects the instantaneous bandwidth of the absorber, in that the larger the L, the smaller the bandwidth. Therefore, it is necessary to ensure that L is set to an appropriate value by optimizing the shape of the AFSS unit cell.

### 3.2. Analysis of the Designed Structure with Different Resistances

**Figure 9:**
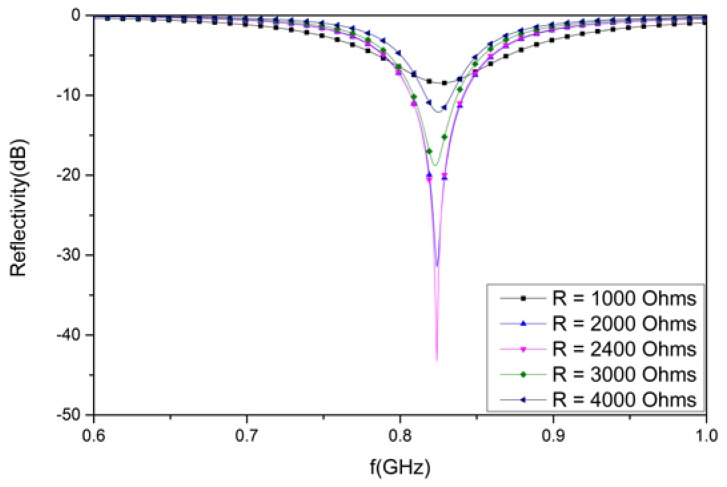
The simulated results for the designed structure with different resistance (R) values.

Figure 9 shows the simulated reflectivity of the proposed absorber with various R and fixed *C_r_* = 1 pF. The reflectivity nadir of the designed structure is −8.5 dB at 827 MHz for R = 1000 Ohms, −31.5 dB at 824 MHz for R = 2000 Ohms, −43.2 dB at 824 MHz for R = 2400 Ohms, −18.8 dB at 823 MHz for R = 3000 Ohms, and −12.1 dB at 825 MHz for R = 4000 Ohms.

The simulated results indicate that R is of great importance for the absorption rate; however, R has little effect on the resonant frequency. The reflectivity nadir first decreases and then increases when R increases from 1000 to 4000 Ohms, and the absorption peak reaches its extreme point when R = 2400 Ohms. Therefore, the simulation analysis for the other parameters in Section 3.3, Section 3.4, Section 3.5 and Section 3.6 is based on the premise that R = 2400 Ohms and *C_r_* = 1 pF.

### 3.3. Analysis of the Designed Structure with Foam Materials of Different Thicknesses

**Figure 10:**
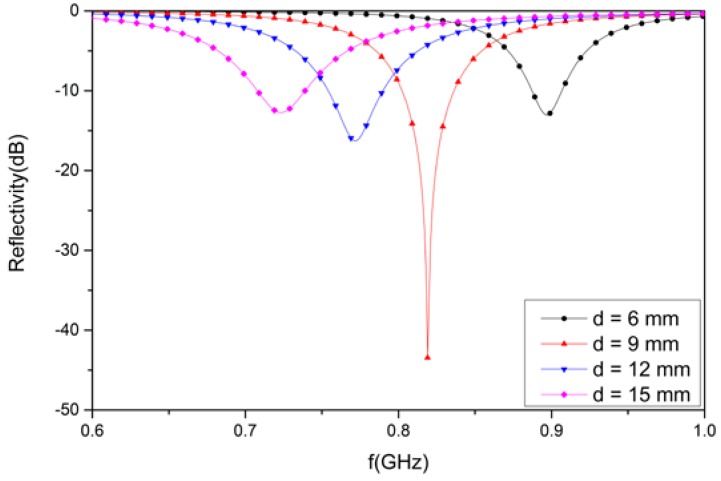
The simulated results for the designed structure with foam materials of different thickness.

Foam materials with different thicknesses were considered in the design structure. To observe the reflectivity, four different thicknesses—6, 9, 12, and 15 mm—were utilized for the proposed design. As observed in Figure 10, the reflectivity of the structure is −13.0 dB at 897 MHz for a 6 mm-thick foam material. Similarly, the value of the reflectivity is −43.2 dB at 824 MHz, corresponding to a 9 mm thickness. In addition, the reflectivity value is −16.3 dB at 772 MHz for a 12 mm-thick foam material. Finally, the reflectivity is −12.7 dB at 723 MHz for a foam thickness of 15 mm. Due to the different thicknesses of the foam material, the reflectivity was different at various resonant frequencies.

The higher the thickness of the substrate materials, the lower the resonant frequency. Further, when the thickness is 9 mm, the reflectivity of the proposed absorber is the lowest, achieving a perfect impedance matching effect.

### 3.4. Analysis of the Designed Structure with Different Thickness FR4 Substrate Materials

**Figure 11:**
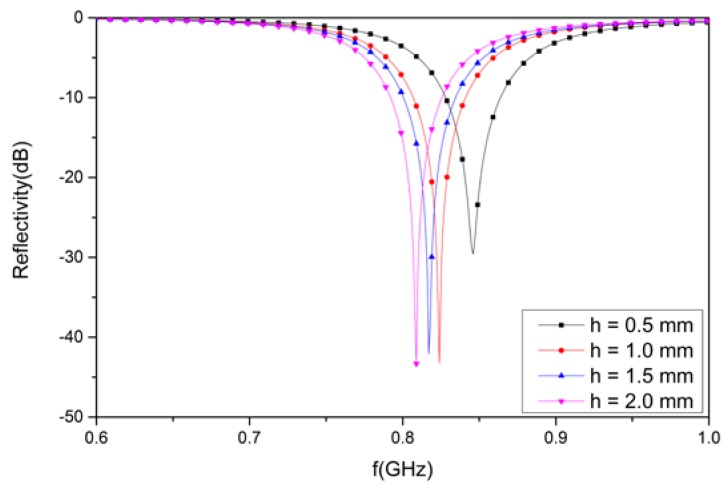
The simulated results for the designed structure with substrate materials of different thicknesses.

Different thicknesses for the substrate materials were considered in the design structure. To observe the reflectivity, four different thicknesses—0.5, 1.0, 1.5, and 2.0 mm—were utilized for the proposed design. It can be observed in Figure 11 that the reflectivity of the structure is −29.6 dB at 846 MHz for a FR4 substrate material with a thickness of 0.5 mm. Similarly, the value of the reflectivity is −43.2 dB at 824 MHz for a 1.0 mm thickness. In addition, the reflectivity value is −42.1 dB at 817 MHz for a 1.5 mm-thick substrate material. Finally, the reflectivity is −43.3 dB at 809 MHz for a thickness of 2.0 mm. Due to the different thicknesses of the substrate material, the reflectivity was different at different resonant frequencies. The higher the thickness of the substrate materials, the lower the resonant frequency.

### 3.5. Analysis of the Designed Structure for Substrate Materials with Varying Permittivity

**Figure 12:**
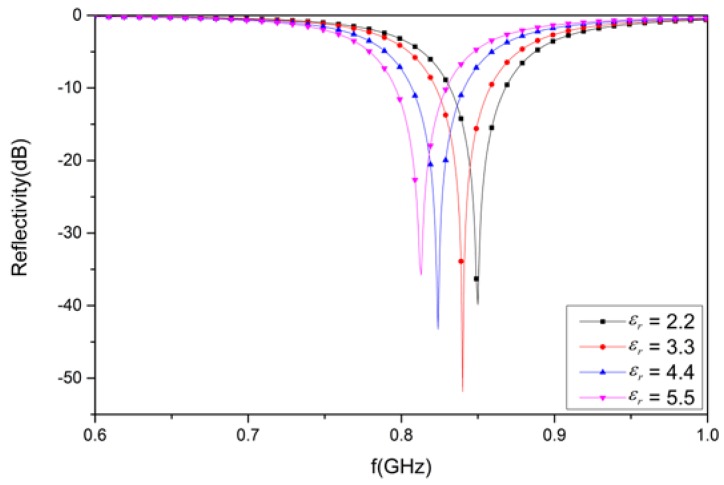
The simulated results for the designed structure with substrate materials of varying permittivity.

Varying permittivity for the substrate materials has an effect on the electromagnetic wave absorption. Different dielectric constants (2.2, 3.3, 4.4, and 5.5) with the same dielectric loss tangent (0.02) were analyzed in the design structure. It can be observed in Figure 12 that the reflectivity of the structure is −39.9 dB at 850 MHz, respectively, for a dielectric constant of 2.2. Likewise, the value of the reflectivity is −51.9 dB at 840 MHz for a dielectric constant of 3.3. In addition, the reflectivity value is −43.2 dB at 824 MHz for a dielectric constant of 4.4, as well as −35.8 dB at 813 MHz for a dielectric constant of 5.5 for the substrate materials.

Due to the varying permittivity of substrate material, the reflectivity was found to be different at different resonant frequencies. A minimum peak was achieved for substrate materials with a dielectric constant of 3.3. However, a maximum peak was achieved for substrate materials with a dielectric constant of 5.5. An obvious variation in the resonant frequency occurs: the higher the permittivity of the substrate material, the lower the resonant frequency.

### 3.6. Analysis of the Designed Structure with Different Incident Angles

The incidence angle (*θ*) was varied from 0 to 60° in steps of 15° to study the reflectivity under oblique incidence. 

**Figure 13:**
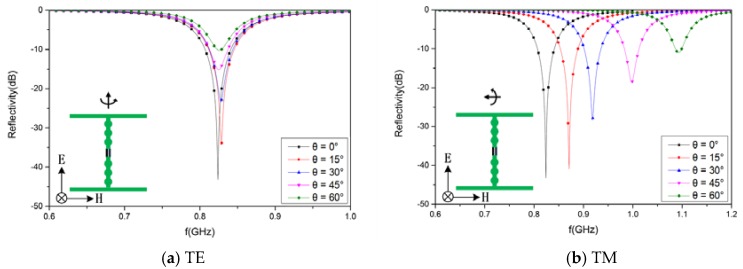
The simulated results for the designed structure with different incident angles: (**a**) TE polarization and (**b**) TM polarization.

It is seen from Figure 13 that the minimum reflectivity values for the design structure are −43.24 dB at 824 MHz for an incidence angle of 0°, −34.3 dB at 829 MHz for 15°, −23.3 dB at 829 MHz for 30°, −15.2 dB at 825 MHz for 45°, and −10.2 dB at 826 MHz for 60° under transverse electric (TE) polarization. The lowest peaks for the reflectivity are −43.2 dB at 824 MHz for an incidence angle of 0°, −41.0 dB at 871 MHz for an incidence angle of 15°, −28.3 dB at 918 MHz for an incidence angle of 30°, −18.6 dB at 998 MHz for an incidence angle of 45°, and −11.0 dB at 1093 MHz for an incidence angle of 60° under transverse magnetic (TM) polarization.

In Figure 13, it is seen that the absorption decreases considerably when increasing the incidence angle under TE polarization, whereas the reduction is smaller for TM polarization, although the resonant frequency shifts rather considerably with *θ*.

Under TE polarization, the electric field is always parallel to the AFSS array, so the impedance (Z) and equivalent inductance (L) of the AFSS is independent of incident angle. Thus, the resonant frequency remains stable with variation of the incident angle. Under TM polarization, there is an angle (*θ*) between the electric field and the AFSS array. The AFSS impedance for TM mode depends on the incident angle [36]. As the incident angle increases, the impedance decreases, and the equivalent inductance L decreases because of Z=jwL. Therefore, as incident angle increases, the resonant frequency will increase for TM polarization.

Under TM polarization, the reflectivity is lowest at 824 MHz for an incidence angle of 0° because of the perfect matched impedance at normal incidence. The reflectivity at 824 MHz increases rapidly to the highest peak with variation of the incident angle because of the gradually mismatched impedance at oblique incidence. This problem can be solved by optimization of the element shape [37,38], which can increase the absorption bandwidth and improve oblique insensitivity.

## 4. Conclusions

A tunable absorber containing an AFSS layer was presented. The AFSS absorber was manufactured by loading resistors and varactors onto an FSS array. A feeding network was designed so that the varactors were connected in parallel. The obtained data show that the operating frequency can be tuned over a wide frequency band from 415 to 822 MHz, while the reverse bias voltage is varied from −5 to −30 V. The results obtained from measurements are in agreement with numerical results. The reflectivity was less than −10 dB, with 90% of the EM energy being absorbed. In addition, the absorbing structure had an ultrathin thickness of 10 mm, which was equivalent to only *λ*/72 of the lower limit frequency. The suggested absorber is appropriate for P-band microwave application.

The simulated results for the electric field and volume loss density distributions indicate that most of the energy losses arise from absorption by the dielectric substrate. A detailed analysis was also carried out on the basis of the AFSS shape, resistor, thickness of the foam, thickness and permittivity of the dielectric substrate, and incident angles to study the tunable absorber design methods.

Compared to other tunable absorbers [39,40], the proposed absorber has the advantage of easy fabrication, an ultrathin thickness, short response time, low absorbing frequency, and wide tunable bandwidth, which makes it promising for smart-skin applications.
